# Wnt/β-catenin controls follistatin signalling to regulate satellite cell myogenic potential

**DOI:** 10.1186/s13395-015-0038-6

**Published:** 2015-04-28

**Authors:** Andrew E Jones, Feodor D Price, Fabien Le Grand, Vahab D Soleimani, Sarah A Dick, Lynn A Megeney, Michael A Rudnicki

**Affiliations:** Regenerative Medicine Program, Ottawa Hospital Research Institute, 501 Smyth Road, Ottawa, ON K1H 8L6 Canada; Department of Cellular and Molecular Medicine, Faculty of Medicine, University of Ottawa, 451 Smyth Road, Ottawa, ON K1H 8M5 Canada; Institut Cochin, Université Paris Descartes, CNRS (UMR 8104), 24 Rue du Fg St Jacques, Paris, France

**Keywords:** Wnt/β-catenin, Skeletal muscle regeneration, Satellite cell, Myogenic differentiation

## Abstract

**Background:**

Adult skeletal muscle regeneration is a highly orchestrated process involving the activation and proliferation of satellite cells, an adult skeletal muscle stem cell. Activated satellite cells generate a transient amplifying progenitor pool of myoblasts that commit to differentiation and fuse into multinucleated myotubes. During regeneration, canonical Wnt signalling is activated and has been implicated in regulating myogenic lineage progression and terminal differentiation.

**Methods:**

Here, we have undertaken a gene expression analysis of committed satellite cell-derived myoblasts to examine their ability to respond to canonical Wnt/β-catenin signalling.

**Results:**

We found that activation of canonical Wnt signalling induces follistatin expression in myoblasts and promotes myoblast fusion in a follistatin-dependent manner. In growth conditions, canonical Wnt/β-catenin signalling prime myoblasts for myogenic differentiation by stimulating myogenin and follistatin expression. We further found that myogenin binds elements in the follistatin promoter and thus acts downstream of myogenin during differentiation. Finally, ectopic activation of canonical Wnt signalling *in vivo* promoted premature differentiation during muscle regeneration following acute injury.

**Conclusions:**

Together, these data reveal a novel mechanism by which myogenin mediates the canonical Wnt/β-catenin-dependent activation of follistatin and induction of the myogenic differentiation process.

**Electronic supplementary material:**

The online version of this article (doi:10.1186/s13395-015-0038-6) contains supplementary material, which is available to authorized users.

## Background

The growth, maintenance, and regeneration of skeletal muscle is attributed to the satellite cell; a mitotically quiescent cell that resides between the basal lamina and sarcolemma of the muscle fiber [1-3]. During muscle regeneration, satellite cells activate, proliferate, and differentiate into new myofibers or fuse to existing myofibers. Skeletal muscle regeneration is a highly orchestrated process contingent upon the proper expression of the paired-box transcription factor Pax7, and the basic helix-loop-helix (bHLH) myogenic regulatory factors (MRFs); Myf5, MyoD, myogenin, and MRF4 [4]. Collectively, MRFs undergo heterodimerization with the ubiquitously expressed E-protein family of bHLH proteins that mediate the recognition of E-box consensus sequences (CANNTG) found in the promoters of many muscle-specific genes [5]. Myf5 and MyoD function to specify myogenic identity in proliferating myoblasts, whereas myogenin and MRF4 are upregulated during differentiation to trigger the expression of myogenic differentiation-specific genes [6].

Skeletal muscle regeneration is regulated through mechanisms involving cellular interactions and extracellular signalling pathways. The Wnt signalling pathway has been shown to play a critical role in regulating various developmental programs through embryonic development and in the regulation of stem cell function in adult tissues [7]. During skeletal muscle regeneration, multiple Wnt signals are activated [8]. We have previously shown that the symmetric expansion of satellite stem cells and their orientation along the axis of division is controlled by the non-canonical Wnt/PCP-pathway [9]. Wnt7a-mediated polarization of α7-integrin and the PCP core-component molecule Vangl2, on opposite poles of the daughter cells, allows both cells to maintain contact with the basal lamina and thus preserve their orientation relative to the niche. However, there are paradoxical roles for the canonical Wnt/β-catenin pathway during adult skeletal muscle regeneration. Evidence exists that suggests Wnt/β-catenin signalling promotes the proliferation and self-renewal of satellite cells, thus preventing myogenic differentiation [10]. In contrast, other research suggests Wnt/β-catenin signalling antagonizes Notch signalling leading to increased terminal differentiation possibly in a BCL9-dependent manner [11,12]. During muscle regeneration, Notch signalling is activated and stimulates the proliferation of satellite cells leading to the expansion of proliferating myoblasts [13]. During differentiation, Wnt/β-catenin signalling antagonizes the effects of Notch signalling, and through reduced GSK3β levels, allows progression of myogenic commitment and differentiation [11]. Among others, we have previously shown that myogenic cells stimulated with Wnt3a activate the canonical Wnt pathway leading to nuclear stabilization of activated β-catenin. *In vivo*, overexpression of Wnt3a in the tibialis anterior (TA) muscle results in an increased number of myofibers, which exhibit a dramatic reduction in the cross-sectional area and reduced regenerative efficiency. *In vitro*, canonical Wnt treatment of satellite cell-derived myoblasts inhibits cellular proliferation, a characteristic not observed by non-canonical Wnt treatment [9]. In addition to modulating the satellite cell compartment, canonical Wnt signalling has been implicated in satellite cell-related transdifferentiation and increasing myogenic potential [14,15].

To further investigate the role of canonical Wnt signalling in adult myogenesis, we analyzed the response of satellite cell-derived myoblasts to Wnt/β-catenin signalling. We show that canonical Wnt signalling is associated with committed satellite cell differentiation potential and control of follistatin expression. Investigation of the role for a Wnt-follistatin pathway leads us to discover that Wnt3a/β-catenin signalling controls the induction of myogenic differentiation via the MyoD-dependent activation of myogenin expression. These findings suggest that Wnt/β-catenin signalling controls multiple steps of adult myogenesis by promoting premature myogenic differentiation in a myogenin-dependent manner.

## Methods

### Cell culture and transfection

Primary myoblasts were isolated from the hind limbs of 4- to 6-week-old wild-type C57Bl/6J mice, as previously described [16], and propagated on collagen-coated culture dishes in Ham’s F10 media supplemented with 20% fetal bovine serum (FBS), 1% penicillin/streptomycin, and 2.5 ng/ml human recombinant bFGF. Myogenic differentiation was induced by shifting to differentiation media (DMEM supplemented with 5% horse serum). Single myofibers were isolated from the extensor digitorum longus (EDL) muscles and cultured in suspension in DMEM supplemented with 15% FBS and 0.5% Chick Embryo Extract (Accurate Chemical Co., Westbury, NY, USA) as previously described [17]. For cell stimulation, recombinant Wnt3a (20 ng/ml), Dickkopf-related protein 1 (Dkk1) (50 ng/ml), and follistatin (250 ng/ml) proteins were added in the culture medium (R&D Systems, Minneapolis, MN, USA). SiRNA transfections were performed in growth media using Lipofectamine 2000 Reagent (Invitrogen, Waltham, MA, USA), as per manufacturer’s instructions. siFollistatin and siMyogenin duplexes were from Ambion (ID s66250, s70334) (Thermo Fisher Scientific, Waltham, MA, USA) and used at the final concentration of 2 nM each.

### Animals

Care of animals is in accordance with institutional guidelines as regulated by the Canadian Council of Animal Care (CCAC). Protocols were approved by Animal Research Ethics Board (AREB) at the University of Ottawa and are reviewed on an annual basis. Eight- to 12-week-old wild-type C57Bl/6J mice were used in this study. The TA muscle was injured by injection of 50 μl cardiotoxin (10 μM, Sigma, St. Louis, MO, USA). (2′Z,3′E)-6-Bromoindirubin-3′-oxime (BIO) was reconstituted in DMSO at a concentration of 5 mM (B1686, Sigma) combined with saline as vehicle and injected directly into the TA muscle in a volume of 10 μl at a final concentration of 1 μM.

### Gene expression analyses

Affymetrix MoGene 1.0 ST chipsets (Santa Clara, CA, USA) were RMA normalized [18] using the xps in Bioconductor R package [19] with the Affymetrix provided MoGene-1_0-st-v1.r4 chip layout and scheme files. The RMA normalized data was log2 transformed and analyzed using the Significance Analysis of Microarrays (SAM) [20] method as implemented in the Bioconductor siggenes package [21]. Heat maps were generated by GenePattern [22]. Gene ontology of expression data was performed using the functional annotation module of DAVID 6.7 [23,24].

### Quantitative PCR

Total RNA was isolated using the RNeasy Kit and subjected to on-column DNase digestion, as per manufacturer’s instructions (Qiagen, Venlo, Netherlands). cDNA synthesis was performed using the Superscript III reverse transcriptase with random hexamer primers (Invitrogen). SYBR Green quantitative polymerase chain reaction (qPCR) was carried out as previously described [25]. Transcript levels were normalized to GAPDH transcript levels. Relative fold change in expression was calculated using the ΔΔCT method (CT values <30). PCR primers were designed using the online Primer3 software (http://primer3.wi.mit.edu) [26]. Primer sequences are listed in Additional file 1: Table S1.

### Western blot

Total protein was harvested in RIPA lysis buffer fortified with protease inhibitors (Complete-Mini; Roche-Boehringer, Ingelheim am Rhein, Germany), and protein concentration was determined by Bradford assay (BioRad, Hercules, CA, USA). Samples (20 μg) were subjected to SDS-PAGE and electroblotted onto Immobilon-P membrane (Millipore, Billerica, MA, USA). Membranes were blocked in 5% nonfat milk in PBST, prior to sequential probing with primary antibody and HRP-conjugated secondary antibody in blocking solution. ECL (Amersham-Pharmacia, Amersham, UK) with BioMax XAR film (Kodak, Rochester, NY, USA) was used to detect target proteins. Primary antibodies used for detection are as follows: α-Pax7 (Developmental Studies Hybridoma Bank, Iowa City, IA, USA), α-myogenin (F5D; Developmental Studies Hybridoma Bank), α-follistatin (Abcam, Cambridge, UK), and α-Tubulin (Sigma). Secondary antibodies were HRP-conjugated anti-mouse and anti-rabbit (1:5000; BioRad).

### Immunocytochemistry

Adherent cell cultures were fixed with 4% PFA, permeabilized with 0.2% Triton-X in PBS, and incubated with primary antibodies: myogenin (M-225) from Santa Cruz (Dallas, TX, USA), α-MyHC (Developmental Studies Hybridoma Bank), and α-Laminin (Sigma). Alexa-488 and Alexa-555 conjugated secondary antibodies that matched the primary antibodies were used at 1:1,000 in PBS (Invitrogen). Nuclei were counterstained with DAPI (Sigma-Aldrich). Images were obtained using an Axioplan2 microscope (Carl Zeiss, Oberkochen, Germany), a ×20 NA 0.75 plan Apochromat (ω/0.17; Carl Zeiss) objective, and a digital Axiocam camera (Carl Zeiss). Digital images were captured using Axiovision (Carl Zeiss) and were processed with Photoshop (Adobe, San Jose, CA, USA).

### ChIP analysis

Protein-DNA complexes were cross-linked with 1% formaldehyde (Sigma) and sheared by sonication. Processing of samples was performed as previously described [27]; 2,000 μg of protein-DNA complexes were immunoprecipitated with 4 to 5 μg of α-myogenin (Santa Cruz) or control Rabbit IgG overnight, followed by wash conditions and DNA elution performed according to the chromatin immunoprecipitation (ChIP) Assay Kit (Upstate Biotechnology, Lake Placid, NY). The immunoprecipitated DNA was subjected to real-time PCR, and results were normalized using control locus representing DNA fragments that were nonspecifically bound. Myogenin chromatin immunoprecipitation sequencing (ChIP-Seq) was performed by chromatin tandem affinity purification, and data was analyzed as described previously [28].

### Statistical analysis

Three or more replicates were analyzed for each experiment presented. Data is shown as standard error of the mean (SEM), and results were assessed for statistical significance by Student’s *T*-test. Differences were considered statistically significant at the *P* < 0.05 level.

## Results

### Canonical Wnt signalling induces follistatin expression in myoblasts

**Figure 1:**
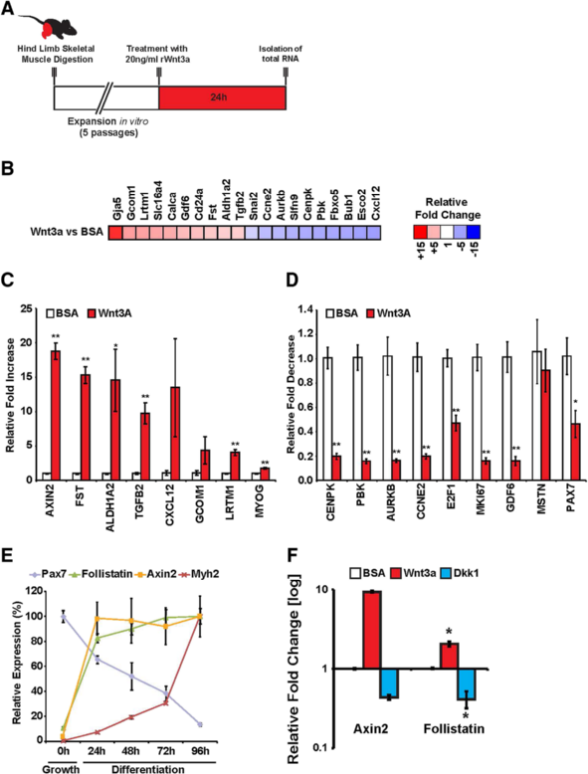
Follistatin expression is regulated by canonical Wnt/β-catenin signalling. **(A)** Schematic depicting the *in vitro* treatment of myoblasts with rWnt3a. **(B)** Global heat map ratio representing the difference in gene expression patterns following treatment with recombinant Wnt3a or control BSA protein. Red represents genes that increase in expression relative to the BSA-treated control, while blue represents a decrease. **(C)** qPCR validation of a subset of genes upregulated following Wnt3a treatment relative to BSA control. Data are presented as the mean ± SEM (*n* = 3, *P < 0.05, **P < 0.01), normalized to GAPDH and shown relative to BSA control. **(D)** qPCR validation of a subset of genes downregulated following Wnt3a treatment relative to BSA control. Data are presented as the mean ± SEM (*n* = 3, *P < 0.05, **P < 0.01), normalized to GAPDH, and shown relative to BSA control. **(E)** qPCR analysis of Pax7, follistatin, Axin2, and Myh2 expression levels in a myoblast differentiation time-course. Pax7 expression is reduced, while Myh2 expression increases during differentiation. Follistatin expression is upregulated during the first 24 h of differentiation in a similar fashion as the Wnt target Axin2. Data are presented as the mean ± SEM (*n* = 3), normalized to GAPDH. **(F)** qPCR analysis of Axin2 and follistatin expression in myoblasts cultured in differentiation media for 24 h, following recombinant Wnt3a, Dkk1, or control BSA protein treatments. Data are presented as the mean ± SEM (*n* = 3), normalized to GAPDH.

Multiple Wnt signals are active during muscle regeneration [8]. To further investigate the role of canonical Wnt signalling in adult myogenesis, we analyzed the response of satellite cell-derived myoblasts to Wnt/β-catenin signalling by genome-wide microarray expression analysis. Low-passage primary myoblasts were treated with recombinant Wnt3a, which we previously demonstrated activates the canonical Wnt/β-catenin pathway in satellite cells [9], or control bovine serum albumin (BSA) protein for 24 h (Figure 1A). Wnt3a stimulation resulted in significant expression changes in 401 genes relative to BSA control (fold change >2, *P* value <0.05); 90 genes were upregulated, while 311 genes were downregulated.

To investigate the global transcriptional effects of Wnt3a treatment, we conducted an unbiased DAVID analysis of our microarray data. Functional annotation clustering of related gene ontology (GO) terms revealed that Wnt3a stimulation strongly regulated cell cycle, cell division, and the progression of mitosis (Additional file 2: Figure S1). Heat maps depicting fold changes for the top 20 regulated genes following Wnt3a treatment relative to BSA-treated controls were generated (Figure 1B). We validated a subset of regulated genes by qPCR and observed upregulation in the expression of the canonical Wnt-responsive gene Axin2 (19-fold), TGFβ2 (approximately tenfold), and Cxcl12 (14-fold) (Figure 1C). Strikingly, we observed that expression of follistatin, a secreted glycoprotein that antagonizes members of the TGF-β superfamily, was increased by 15.3-fold following Wnt3a stimulation (Figure 1C), independent of an effect on myostatin expression (Figure 1D). We observed a decrease in the expression of genes associated with the progression of mitosis and cell proliferation E2F1 (approximately twofold), Ki67 (sixfold), and cyclin E2 (fivefold) following Wnt3a stimulation (Figure 1D). Furthermore, following Wnt3a stimulation Pax7 expression was reduced (approximately twofold) while myogenin was increased (approximately twofold), indicative of myogenic commitment (Figure 1C,D). Following our global microarray analysis, we focused on follistatin, given its strong upregulation following Wnt treatment and its previously characterized role in promoting differentiation and muscle hypertrophy [29-31].

To further study follistatin stimulation in response to activation of Wnt/β-catenin signalling, we analyzed its relative expression during a myoblast differentiation time course (Figure 1E). During myogenic differentiation, Pax7 transcription diminishes while the expression of differentiation markers (such as myosin heavy chain, Myh2) become elevated. We observed that Axin2 expression was strongly increased within the first 24 h of differentiation, consistent with an increase in Wnt/β-catenin signalling, and this was concomitant to a tenfold increase in follistatin expression.

Given follistatin expression is upregulated following the induction of differentiation, we next examined if the modulation of Wnt/β-catenin signalling during myogenic differentiation resulted in further variation in follistatin expression. To this aim, we treated differentiating cells with recombinant Wnt3a protein, the canonical Wnt antagonist Dkk1, or BSA as a control. We observed that while Wnt3a stimulation resulted in an increase in follistatin and Axin2 expression levels, their expression levels were reduced following Dkk1-mediated inhibition of Wnt/β-catenin activity (Figure 1F). These observations further suggest that the regulation of follistatin expression following Wnt3a treatment is dependent on the canonical Wnt signalling pathway.

### Canonical Wnt signalling promotes myoblast fusion in a follistatin-dependent manner

**Figure 2:**
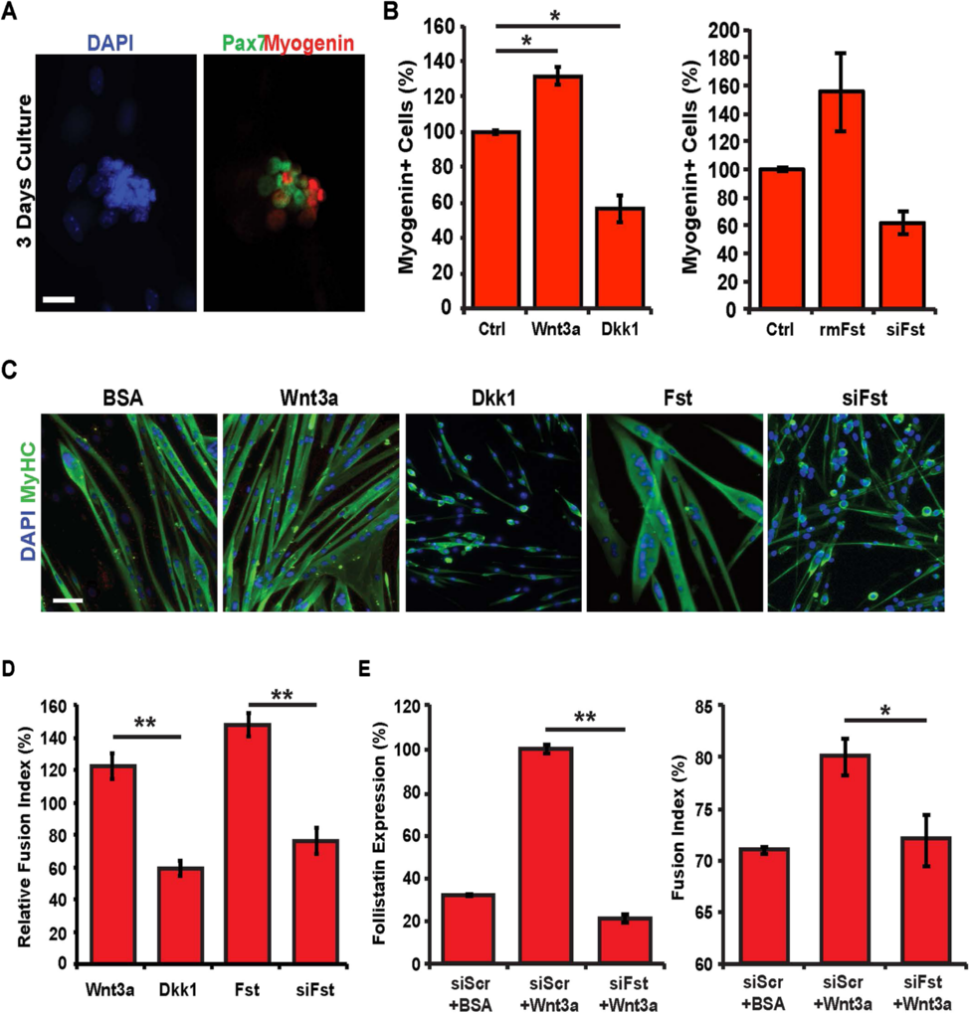
Canonical Wnt/β-catenin signalling promotes myoblast fusion in a follistatin-dependent manner. **(A)** EDL single myofibers were cultured in floating conditions for 3 days, and immunostained for Pax7 and myogenin expression. Shown is a typical myogenic cell cluster. **(B)** Percentage of myogenin+ cells on myofibers following 24 h of recombinant Wnt3a, Dkk1, or control BSA protein treatments (left) and following 24 h recombinant follistatin protein treatment, siRNAs against follistatin transfection, or control scrambled siRNAs transfection. **(C)** Myoblasts were cultured in differentiation medium for 3 days in the presence of recombinant Wnt3a, Dkk1, follistatin, or control BSA proteins or transfected with siRNAs against follistatin and immunostained for Myh2 expression. Scale bar 25 μM. **(D)** Quantification of the fusion index in myogenic cultures. Changes are expressed as relative to the control (BSA treatment or siScr transfection). Data are presented as the mean ± SEM (*n* = 3, **P < 0.01). **(E)** qPCR analysis of follistatin expression (left) and quantification of the fusion index (right) of myoblasts cultured in differentiation media for 24 h treated with siScr and BSA, siScr and Wnt3a, or siFst and Wnt3a. Data are presented as the mean ± SEM (*n* = 3, *P < 0.05, **P < 0.01), normalized to GAPDH.

To determine if the induction of follistatin expression by canonical Wnt signals was necessary for myogenic differentiation, we conducted gain- and loss-of-function experiments. We cultured single EDL myofibers in floating conditions and assessed the proportion of differentiating myogenin+ cells within clusters after 3 days *in vitro* (Figure 2A). We observed that treatment of either Wnt3a or follistatin proteins after 24 h in culture resulted in a 32% ± 5% increase in the proportion of myogenin+ satellite cells, whereas a reduction in myogenin+ satellite cells was observed following Dkk1 protein stimulation (43% ± 8%), or knockdown of follistatin expression via siRNA (38% ± 8%) when compared to controls (untreated cells or cells transfected with non-silencing *Scrambled* siRNAs) (Figure 2B). Further, we observed that either Wnt3a or follistatin treatment resulted in larger myotubes with an increased number of nuclei per myotube, while inhibition of Wnt/β-catenin signalling via Dkk1 treatment and inhibition of follistatin signalling via siRNA-mediated gene silencing limited myogenic fusion, and led to the formation of short myotubes (Figure 2C,D).

To validate that follistatin acts downstream of Wnt/β-catenin signalling during myogenic differentiation, we first determined the optimal concentration of siRNA against follistatin for transfection in the presence of Wnt3a to reduce follistatin expression to control levels (transfected with non-silencing Scrambled siRNAs) (Figure 2E, left). We next observed that in differentiating myoblasts, the increase in fusion index induced by Wnt3a is dependent on the increase in follistatin expression (Figure 2E, right). Taken together, these observations implicate follistatin as a canonical Wnt signalling target whose expression promotes differentiation-induced myogenic fusion.

### Canonical Wnt/β-catenin signalling primes myoblasts for myogenic differentiation

**Figure 3:**
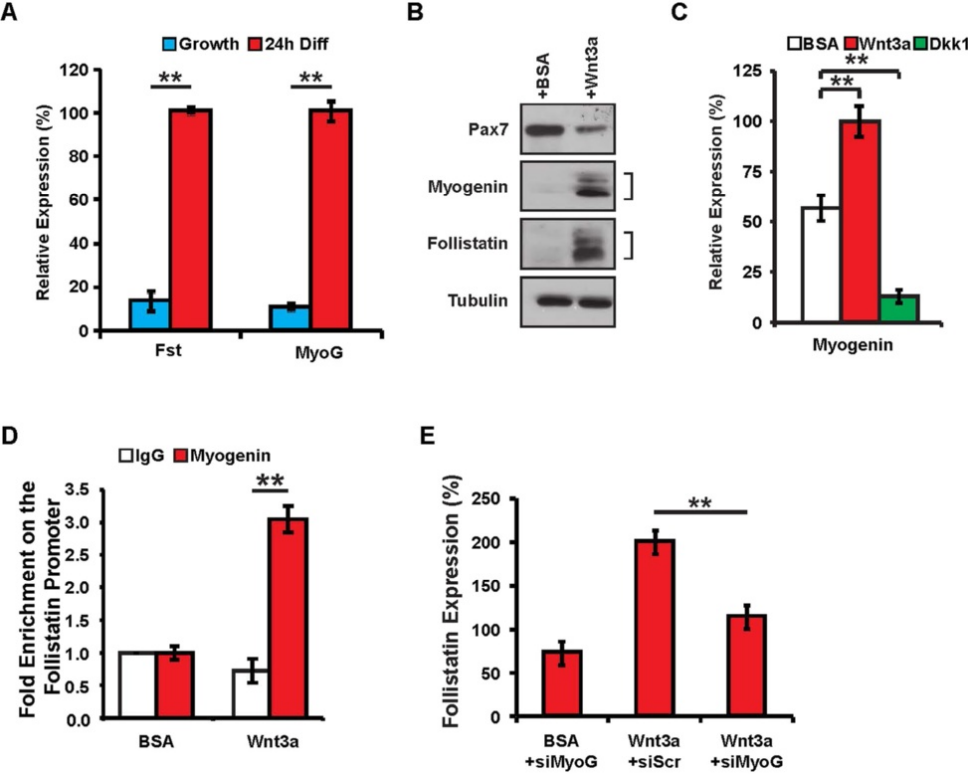
Wnt3a prime myoblasts for myogenic differentiation. **(A)** qPCR analysis of follistatin and myogenin expression levels in proliferating and differentiating myoblasts. Data are presented as the mean ± SEM (*n* = 3, **P < 0.01), normalized to GAPDH. **(B)** Western blot analysis of Pax7, myogenin, and follistatin expression in proliferating myoblasts following recombinant Wnt3a or control BSA protein treatments. **(C)** qPCR analysis of myogenin expression in proliferating myoblasts following recombinant Wnt3a, Dkk1, or control BSA protein treatments. Data are presented as the mean ± SEM (*n* = 3, **P < 0.01), normalized to GAPDH. **(D)** qPCR analysis of locus enrichment in chromatin immunoprecipitated with α-myogenin or control IgG antibodies from proliferating myoblasts following recombinant Wnt3a or control BSA protein treatments. Data are presented as the mean ± SEM (*n* = 3, **P < 0.01). **(E)** qPCR analysis of follistatin expression in myoblasts treated with siScr and BSA, siScr and Wnt3a, or siMyog and Wnt3a. Data are presented as the mean ± SEM (*n* = 3, **P < 0.01), normalized to GAPDH.

A recent report demonstrated that myogenin expression represses genes involved in cell cycle progression, and is sufficient to induce exit from the cell cycle [32]. Since we observed a major suppression of cell proliferation genes following Wnt3a stimulation (Figure 1D, Additional file 2: Figure S1), we sought to explore the possible involvement of myogenin during Wnt/β-catenin induced muscle differentiation. We first quantified the relative expression levels of follistatin and myogenin in growth conditions, where expression levels are low relative to differentiation conditions (Figure 3A). Following Wnt stimulation, we observed increased myogenin and follistatin and decreased Pax7 protein levels in growth conditions relative to BSA controls (Figure 3B). While expressions of myogenin and follistatin are induced during differentiation, it was striking to observe that Wnt3a stimulation upregulated their expression in growth conditions.

Previous data revealed that β-catenin directly interacts with MRFs enhancing their ability to bind E-box regulator elements and ultimately promoting muscle differentiation [33]. More recent evidence demonstrates that β-catenin can directly bind and activate cis elements of the MyoD promoter [34]. In light of β-catenin’s ability to interact with MyoD and myogenin and enhance their capacity to activate downstream target genes, we examined a recently unpublished data set comprising myogenin chromatin immunoprecipitation coupled with massive parallel high-throughput sequencing (ChIP-Seq), which allowed us to identify a myogenin regulatory element upstream of the follistatin promoter during differentiation. We identified a conserved regulatory E-box element 30-kb upstream of the follistatin gene locus that is occupied by myogenin during early differentiation (Additional file 3: Figure S2, *unpublished data*). Interestingly, a recent publication from our lab identifies this element as being occupied during proliferation not by myogenin but rather by the Snail-HDAC1/2 repressive complex inhibiting myogenin from accessing this E-box motif [28]. We further analyzed the binding of myogenin on this regulatory element in proliferating conditions following Wnt3a stimulation by ChIP-PCR. Our results demonstrate an approximately threefold enrichment of myogenin binding to this conserved element following Wnt3a stimulation (Figure 3D). Taken together, these observations link canonical Wnt signalling to the expression of follistatin in a myogenin-dependent manner.

### Follistatin acts downstream of myogenin during differentiation

To demonstrate that follistatin acts downstream of myogenin expression, we first determined the optimal concentration of siRNA against myogenin for transfection in the presence of Wnt3a to reduce myogenin expression similar to control levels (BSA treatment transfected with non-silencing Scrambled siRNAs) (Additional file 4: Figure S3). Wnt3a treatment and subsequent delivery of siMyoG resulted in an 86% ± 3% reduction in follistatin expression in proliferating myoblasts (Figure 3E). These results suggest that, *in vitro*, canonical Wnt stimulation of follistatin occurs in a myogenin-dependent manner and leads to precocious myogenic differentiation.

### Canonical Wnt signalling promotes premature differentiation *in vivo*

**Figure 4:**
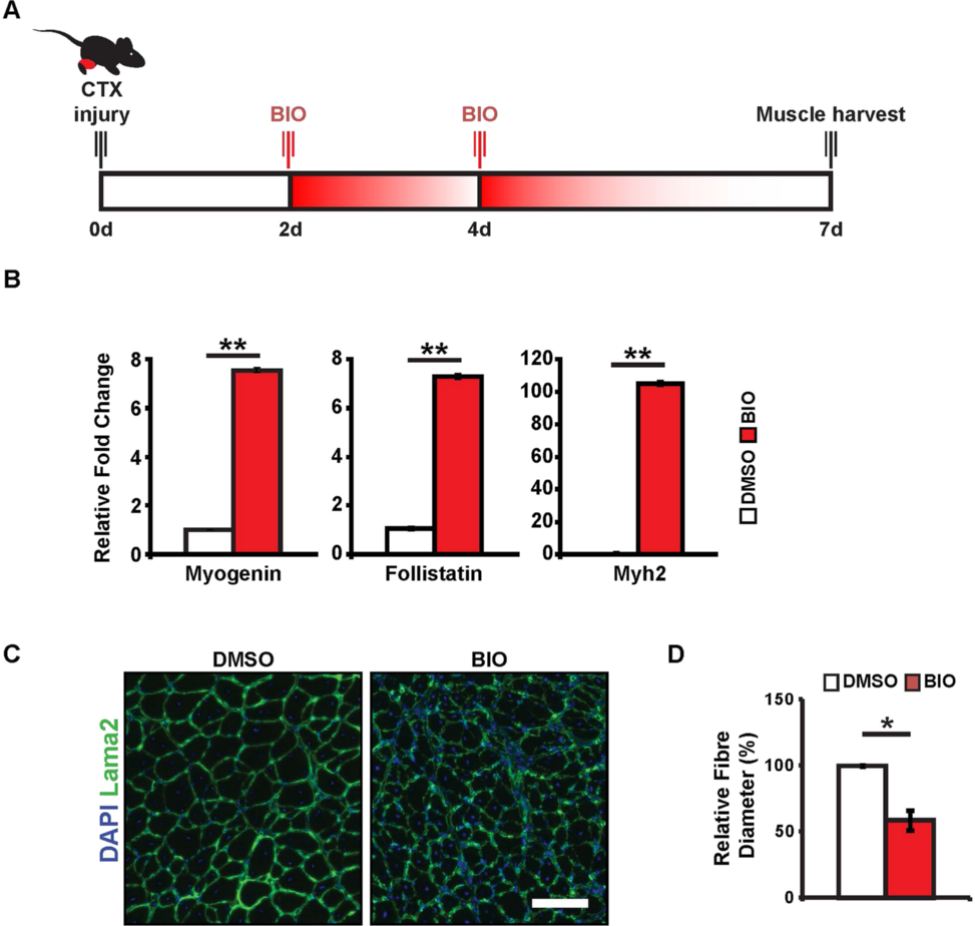
Canonical Wnt signalling promotes precocious differentiation *in vivo.* **(A)** Experimental time-course for *in vivo* muscle injury. Mice undergo direct muscle injections of BIO 2 and 4 days post-CTX injury. **(B)** qPCR analysis of myogenin, follistatin, and Myh2 expression in TA muscle 7 days following CTX injury. Data are presented as the mean ± SEM (*n* = 3, **P < 0.01), normalized to GAPDH. **(C)** Representative cryosections of TA muscle 7 days following CTX injury and treatment with GSK3β inhibitor (BIO) or DMSO control. Scale bar 50 μm. **(D)** Quantification of muscle fiber caliber in TA muscles 7 days following CTX injury and BIO treatment. Data are presented as the mean ± SEM (*n* = 3, *P < 0.05).

To investigate the role of the Wnt-follistatin pathway in muscle regeneration *in vivo*, we treated regenerating muscle with a small molecule agonist of canonical Wnt signalling (Figure 4A). Previous studies demonstrate that Wnt proteins are secreted by the myofiber during muscle regeneration [9,11,15]. To further examine Wnt/β-catenin signalling during regeneration, we treated regenerating TA muscle at 2 and 4 days following cardiotoxin (CTX) injury with either DMSO vehicle or BIO, a pharmacological inhibitor of GSK3β activity [35]. By inhibiting GSK3β, we effectively activate the canonical Wnt signalling pathway in satellite cells during the core stages of muscle regeneration. Seven days following injury, we harvested RNA from total muscle tissue and examined gene expression profiles. Consistent with our *in vitro* data, we observed an overall increase in myogenin and follistatin expression, as well as an increase in Myh2 (Figure 4B). These results suggest that *in vivo* stimulation of β-catenin activity promotes myogenic differentiation.

Following the direct delivery of the small molecule Wnt agonist BIO, we observed altered muscle fiber morphology (Figure 4C), resulting in an increased number of fibers, as well as a dramatic reduction in cross-sectional area (Figure 4D). These results corroborate previous experiments examining Wnt stimulation in regenerating muscle [9,11]. Furthermore, these results substantiate our *in vitro* observations as treatment of regenerating skeletal muscle with a canonical Wnt signalling agonist reduces its regenerative potential through initiating premature myogenic differentiation. Taken together, these observations suggest canonical Wnt signalling promotes differentiation by stimulating the expression of myogenin and follistatin *in vivo*.

## Discussion

Canonical Wnt signalling is essential for proper myogenic development and regeneration. However, in aged skeletal muscle, excessive Wnt/β-catenin signalling results in precocious differentiation of satellite cells into the fibroblast lineage, resulting in a depletion of the satellite cell pool and inefficient muscle regeneration [11,14]. Furthermore, recent studies have shown that canonical Wnt/β-catenin agonists promote myogenic differentiation [36-38].

Interestingly, we observed that Wnt3a promoted cell cycle arrest and activation of myogenin - a core MRF transcription factor responsible for fusion and terminal differentiation (Figure 1). *In situ* analysis of satellite cells on single myofibers identified an increase in the percentage of satellite myogenic progenitors that express myogenin. Following Wnt3a treatment myoblasts induced to differentiate displayed enhanced fusion (Figure 2B,C), an effect lost following Dkk1-mediated inhibition of endogenous Wnt signalling. Endogenous Wnt3a expression is induced in cultured myofibers, consistent with the observation that Wnt expression is low during the early phase of regeneration when progenitor expansion is occurring, with a subsequent increase to promote myogenic commitment and differentiation [11,15,39]. Interestingly, recent research suggests that it is not the initial activation of Wnt signalling but rather it is inhibition following the expansion of the satellite cell pool that is critical for proper adult skeletal muscle regeneration [40].

Among the genes upregulated following Wnt3a stimulation of proliferating myoblasts, the myogenic regulatory factor myogenin plays a prominent role in regulating myoblast fusion and terminal differentiation. While myogenin expression is normally upregulated during differentiation, we observed a strong increase of myogenin protein following Wnt3a stimulation (Figure 3B), a feature not observed following non-canonical Wnt7a stimulation (data not shown). This upregulation of myogenin may serve as a node for the canonical Wnt-induced switch to myogenic differentiation, regardless of the pro-proliferative culture conditions. We previously observed that canonical Wnt signalling reduced myoblast proliferation rate *in vitro* [9], and these results are further explained taking into account the activation of myogenin and the significant repression of genes involved in cell cycle progression (Figure 1C, Additional file 5: Table S2).

Myogenic differentiation is a highly orchestrated process, with cell cycle withdrawal occurring before the expression of contractile proteins or cell fusion [41,42]. Myogenin expression leads to the downregulation of genes involved in cell cycle progression, and its expression is sufficient to induce cell cycle withdrawal [32]. This indirect cell cycle repression occurs via the myogenin-dependent expression of miR-20a, a microRNA well characterized in its silencing of E2F1 and E2F3 [43,44]. In addition, the induction of myogenin expression is dependent on tissue-specific expression of MyoD, co-operating with ubiquitously expressed Six, Mef2, and Pbx1 protein families and in concert with the epigenetic modifications of chromatin structure [45]. While Wnt3a treatment did not further induce MyoD expression (data not shown), it should be noted that activated myoblasts already express high levels of MyoD protein.

While its transcriptional activity is generally associated with TCF/Lef proteins, recent studies are establishing a potential TCF/Lef-independent function of β-catenin. As discussed, MyoD is able to complex with β-catenin independent of E-proteins [33], whose interaction preferentially binds muscle-specific E-box elements and promotes myogenic differentiation. In addition to MyoD, β-catenin has been described in regulating TCF-independent transcription with Sox proteins [46], FoxO proteins [47], homeodomain proteins Prop1 [48] and PitX2 [49], hypoxia-inducible factor 1α (HIF1α) [50], and type I and type II nuclear receptors [51]. These findings further suggest that while the classical TCF/Lef-dependent β-catenin transcriptional activation remains important, a TCF/Lef-independent role for β-catenin provides a previously uncharacterized level of gene regulation.

Follistatin is a secreted glycoprotein that antagonizes various members of the TGF-β superfamily, including myostatin [52,53]. Originally identified in porcine ovarian follicular fluid, follistatin was suggested to promote muscle fiber hypertrophy by antagonizing the myostatin mediated repressive effects on myogenic progenitor differentiation and muscle fiber growth [29,30]. Through inhibiting myostatin and activin A activity, follistatin overexpression leads to a more dramatic muscle phenotype than the myostatin-null mouse [31]. Strikingly, follistatin regulation remains functional in myostatin-null mice, suggesting a myostatin-independent function [54,55]. Recent observations suggest that follistatin promotes muscle hypertrophy through suppressing the phosphorylation of Smad3, resulting in potentiation of mTOR/S6 protein kinase (S6K)/S6 ribosomal protein (S6RP) signalling and inhibition of GSK3β [56,57]. The mTOR-dependent phosphorylation of S6RP leads to increased protein synthesis and cell size from enhanced protein translation initiation and elongation [58].

In the absence of exogenous canonical Wnt stimulation, myogenin is bound to the follistatin locus early during differentiation and is presumably responsible for the rapid follistatin expression observed following 24 h of myogenic differentiation (Figure 1D). We previously found that the Snail DNA-binding zinc finger transcriptional repressors bind the same DNA motif as the bHLH transcription factors MyoD and myogenin [28]. In proliferating myoblasts, Snail prevents MyoD occupancy on differentiation-specific regulatory elements, and the change from Snail to MyoD/myogenin binding often results in enhancer switching during differentiation and recruitment of histone acetylase activity on the myogenin promoter.

Upon Wnt3a stimulation, we observe myogenin binding to an E-box element not occupied during proliferation conditions (Figure 3D, Additional file 2: Figure S1). While TCF/Lef sites have been identified in the follistatin promoter [59,60], our data suggests that myogenin is directly involved in regulating follistatin transcription, through a regulatory E-box element, both during differentiation and following Wnt3a-induced precocious differentiation.

Following injury-induced regeneration, we observed that treatment with a GSK3β antagonist further potentiated the differentiation effects of canonical Wnt signalling (Figure 4B,C). Specifically, further induction of myogenic genes and follistatin expression induced rapid differentiation of regenerating fibers, resulting in premature fusion and production of an increased number of smaller fibers (Figure 4C,D). The application of a pharmacological antagonist, which replicates observations from plasmid electroporation and protein injections, remains promising as it provides efficient delivery and dispersion within structured skeletal muscle.

## Conclusions

Canonical Wnt/β-catenin signalling promotes myogenic differentiation. The mechanism by which this occurs was previously thought to involve direct binding of β-catenin to the follistatin promoter [36]. Our experiments suggest a complementary pathway exists involving Wnt/β-catenin induction of myogenin and its subsequent binding to and activating the expression of follistatin. These results provide a novel mechanistic framework that helps explain how canonical Wnt/β-catenin signalling promotes early myogenic differentiation.

## Additional files

Additional file 1: Table S1. Primer sequences used for gene-specific mRNA quantitation by qPCR. Additional file 2: Figure S1. Enriched gene ontology terms following Wnt3a treatment. (A) Gene ontology terms enriched among upregulated genes following Wnt3a stimulation for 24 h relative to BSA control. (B) Gene ontology terms enriched among downregulated genes following Wnt3a stimulation for 24 h relative to BSA control. Additional file 3: Figure S2. Myogenin binding site upstream of the follistatin locus. Myogenin peaks mapped to the UCSC genome browser, with scale set to automatic. A myogenin bound motif 30-kb upstream of the follistatin promoter is shown following induction of 2 days differentiation (Rudnicki Lab, unpublished data). Additional file 4: Figure S3. Wnt3a activates myogenin expression in proliferating myoblasts myogenin expression levels analyzed by qPCR in proliferating myoblasts treated for 24 h with siMyog and BSA, siScr and Wnt3a, or siMyog and Wnt3a. Data are presented as the mean ± SEM (*n* = 3, ***P* < 0.01), normalized to GAPDH. Additional file 5: Table S2. Summary of Wnt3a regulated changes in gene expression as assessed by Affymetrix microarray performed in biological triplicate.

## Abbreviations

bFGF: basic fibroblast growth factor
bHLH: basic helix-loop-helix
BIO: (2′Z,3′E)-6-bromoindirubin-3′-oxime
BSA: bovine serum albumin
ChIP: chromatin immunoprecipitation
CTX: cardiotoxin
Dkk1: Dickkopf-related protein 1
EDL: extensor digitorum longus
FBS: fetal bovine serum
GSK3: glycogen synthase kinase 3
HDAC: histone deacetylase
MyHC: myosin heavy chain
Myh2: myosin heavy chain 2
qPCR: quantitative polymerase chain reaction
TA: tibialis anterior
