# Supplementary material for: Wnt/β-catenin controls follistatin signalling to regulate satellite cell myogenic potential
Source: Skelet Muscle. 2015 Apr 28;5:14. doi: 10.1186/s13395-015-0038-6 (PMC4421991; doi:10.1186/s13395-015-0038-6)
Supplement: Additional file 1: Table S1. — Primer sequences used for gene-specific mRNA quantitation by qPCR. [file 13395_2015_38_MOESM1_ESM.pdf]

Table S1. Primer Sequences

Real Time RT-PCR	
Gja5_F	CAGAGCCTGAAGAAGCCAAC
Gja5_R	GCAACCAGGCTGAATGGTAT
Gcom_F	TTGTGGACGTGACTTTGGAA
Gcom_R	ATTGGCTTCTTGCGATGACT
Lrtm1_F	ACCCTTGGATTTGTGACTGC
Lrtm1_R	AGAACCAGGCTGCTGGACTA
Gdf6_F	CAATGCCAGCTTTTTCCAGT
Gdf6_R	CCCACCAGCTCTTCTTTGTC
Fst_F	GCTCCTGCTGCTGCTACTCT
Fst_R	CCCGTTGAAAATCATCCACT
Aldh1a2_F	CCATGACTTCCAGCGAGAT
Aldh1a2_R	CAGGGAACACTCTCCCACTC
Tgfb2_F	CCGGAGGTGATTTCCATCTA
Tgfb2_R	GCGGACGATTCTGAAGTAGG
Ccne2_F	GGCATGTTCACAGGAGGTTT
Ccne2_R	AATCCCAATGAGTTGAAGCA
E2F1_F	GACTCCTCGCAGATCGTCAT
E2F1_R	CAGCGAGGTACTGATGGTCA
Mki67_F	CAGACTTGCTCTGGCCTACC
Mki67_R	TGTCCACCAAAGGATACACG
Cenpk_F	CATTTTCCTCTGCCTGAAGC
Cenpk_R	TATGGTGGCCAAAAGGAATC
Pbk_F	GTGGAAGTCCTTTTCCAGCA
Pbk_R	TTCATCCAATGGCAGAGAGA
Aurkb_F	AGGTCTGCAGGGAGAACTGA
Aurkb_R	ACGTCTCACTGTGGCTAGGG
Axin2_F	CGACCTCAAGTGCAAACTCT
Axin2_R	CTGGATAACTCGCTGTCGTT
Myog_F	GAAGTGAATGAGGCCTTCG
Myog_R	ACGATGGACGTAAGGGAGTG
Mstn_F	GACAGCAGTGATGGCTCTTT
Mstn_R	TAGGAGTCTTGACGGGTCTG
Pax7_F	CTGGATGAGGGCTCAGATGT
Pax7_R	GGTTAGCTCCTGCCTGCTTA
Myh2_F	CAGAACAGAGACGGCTTCAT
Myh2_R	AGTTTCTCCCCAAACATCGT
GAPDH_F	TGTCCGTCGTGGATCTGAC
GAPDH_R	GGTCCTCAGTGTAGCCCAAG
ChIP	
GAPDH(-3kb)_F	TCCAGTGAGGACGGTATGAT
GAPDH(-3kb)_R	CATAAAGATGGGGCAAAATG
Fst(-30kb)_F	CTTTGTGCCCGTTTGAAAAT
Fst(-30kb)_R	GAAAGTGCCGCAAAGAAGAG
