# Supplementary figures and images for: Wnt/β-catenin controls follistatin signalling to regulate satellite cell myogenic potential
Source: Skelet Muscle. 2015 Apr 28;5:14. doi: 10.1186/s13395-015-0038-6 (PMC4421991; doi:10.1186/s13395-015-0038-6)

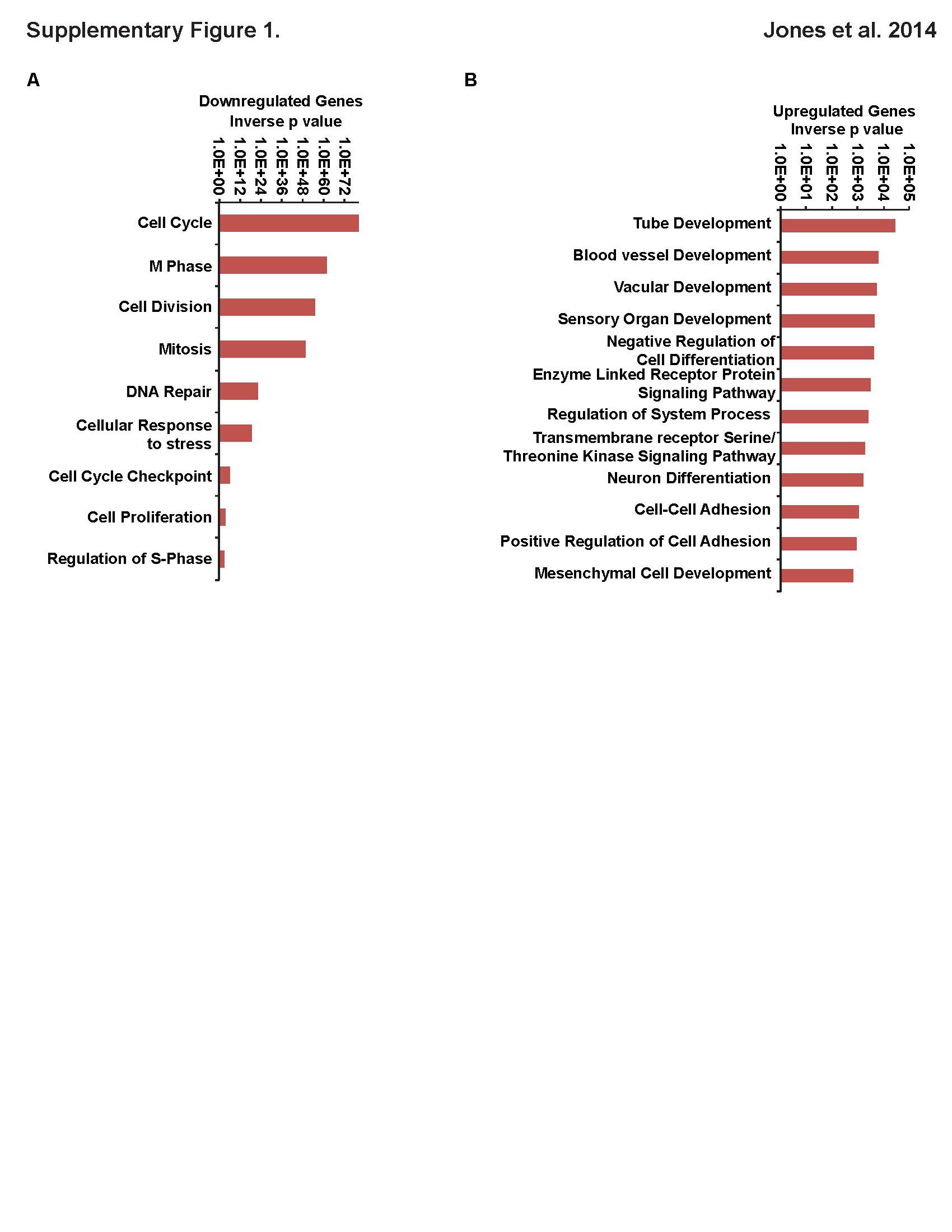

Supplement: Additional file 2: Figure S1. — Enriched gene ontology terms following Wnt3a treatment. (A) Gene ontology terms enriched among upregulated genes following Wnt3a stimulation for 24 h relative to BSA control. (B) Gene ontology terms enriched among downregulated genes following Wnt3a stimulation for 24 h relative to BSA control. [file 13395_2015_38_MOESM2_ESM.jpeg]

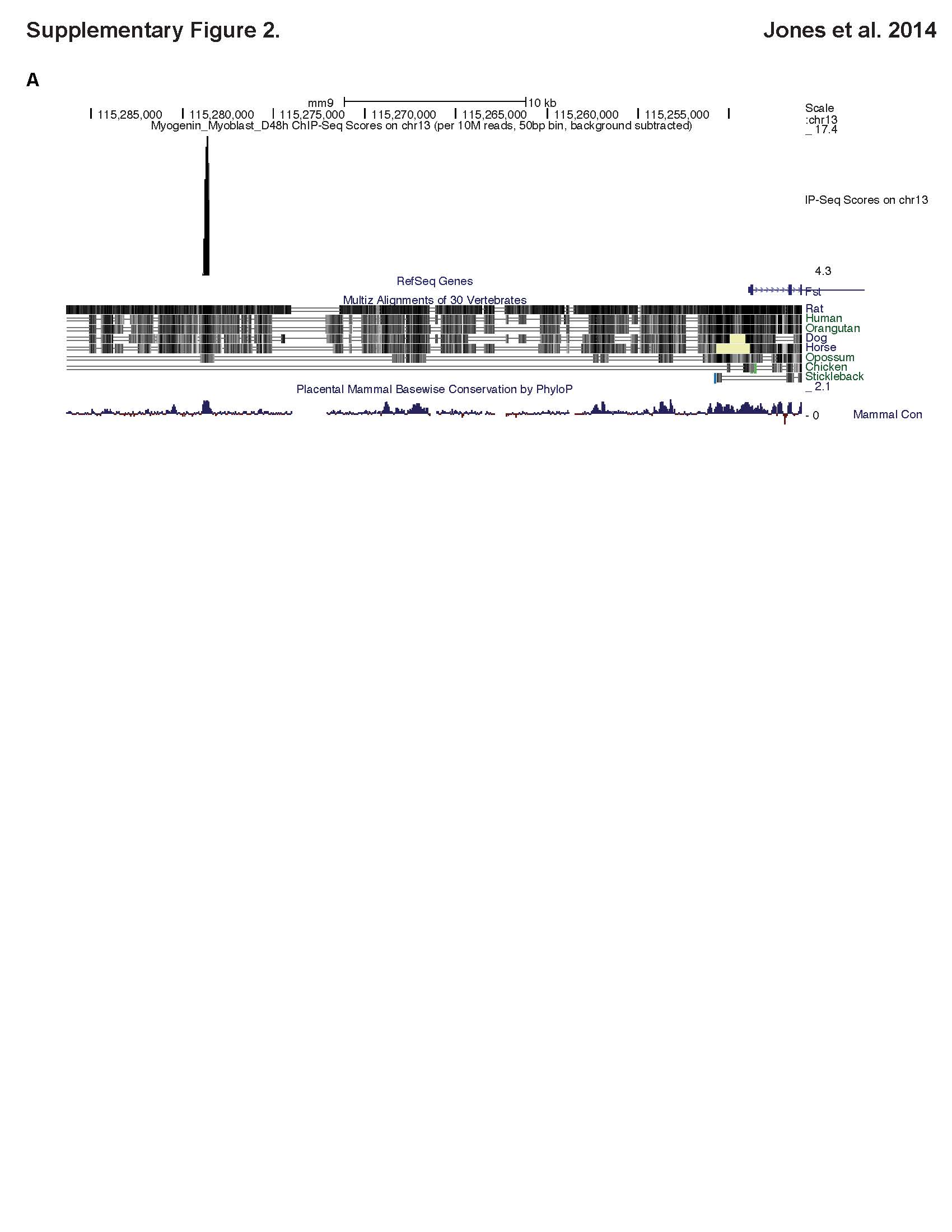

Supplement: Additional file 3: Figure S2. — Myogenin binding site upstream of the follistatin locus. Myogenin peaks mapped to the UCSC genome browser, with scale set to automatic. A myogenin bound motif 30-kb upstream of the follistatin promoter is shown following induction of 2 days differentiation (Rudnicki Lab, unpublished data). [file 13395_2015_38_MOESM3_ESM.jpeg]

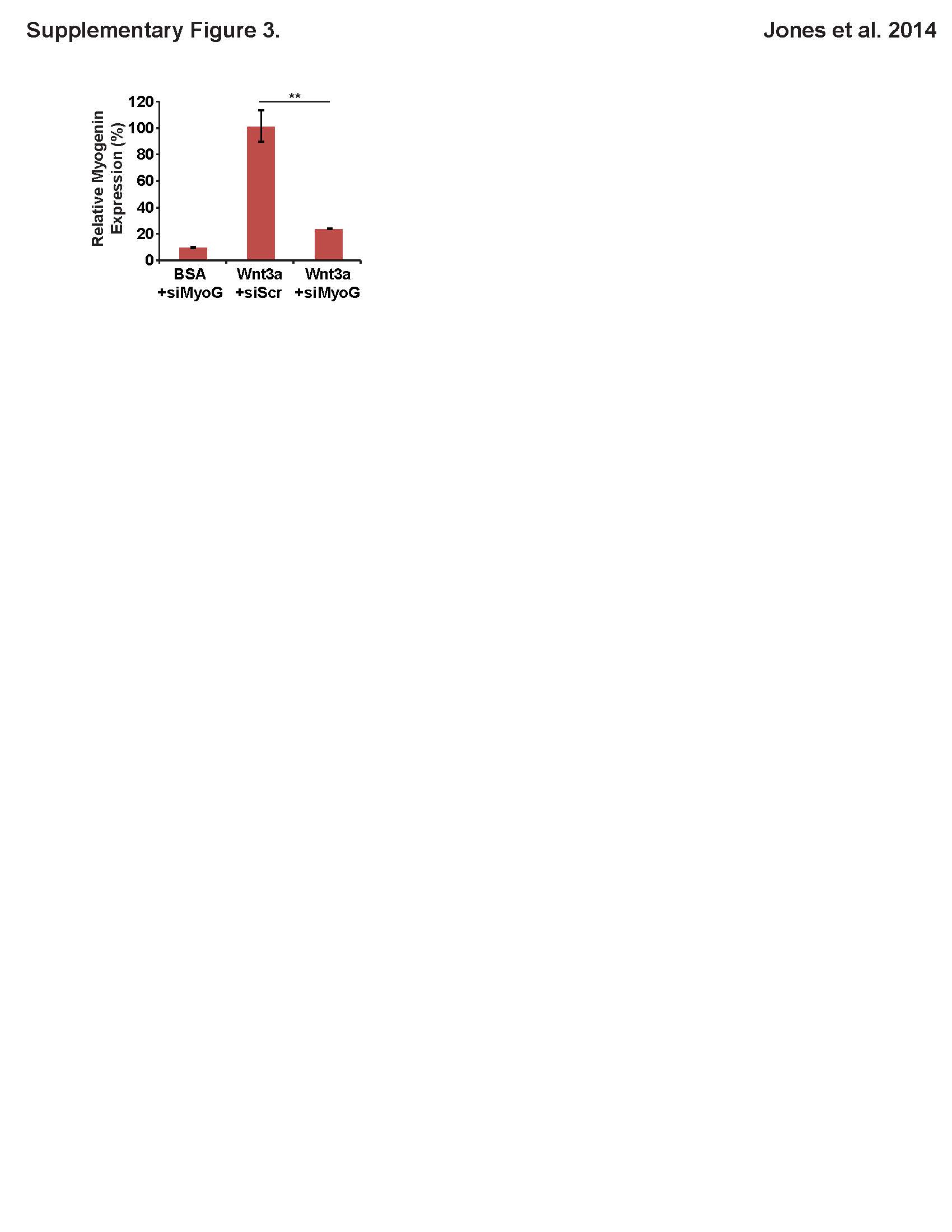

Supplement: Additional file 4: Figure S3. — Wnt3a activates myogenin expression in proliferating myoblasts myogenin expression levels analyzed by qPCR in proliferating myoblasts treated for 24 h with siMyog and BSA, siScr and Wnt3a, or siMyog and Wnt3a. Data are presented as the mean ± SEM (n = 3, **P < 0.01), normalized to GAPDH. [file 13395_2015_38_MOESM4_ESM.jpeg]
