# Supplementary material for: Wnt/β-catenin controls follistatin signalling to regulate satellite cell myogenic potential
Source: Skelet Muscle. 2015 Apr 28;5:14. doi: 10.1186/s13395-015-0038-6 (PMC4421991; doi:10.1186/s13395-015-0038-6)
Supplement: Additional file 5: Table S2. — Summary of Wnt3a regulated changes in gene expression as assessed by Affymetrix microarray performed in biological triplicate. [file 13395_2015_38_MOESM5_ESM.pdf]

Table S2. Wnt3a Regulated Changes in Gene Expression

Gene Symbol	Ensembl ID	Fold Change	P-value
Gja5	ENSMUSG00000057123	12.5375	7.34E-42
Gcom1	ENSMUSG00000041361	6.2437	5.06E-28
Lrtm1	ENSMUSG00000045776	6.0716	5.92E-36
Slc16a4	ENSMUSG00000027896	5.8579	3.77E-33
Calca	ENSMUSG00000030669	5.4211	1.46E-32
Gdf6	ENSMUSG00000051279	4.5604	1.43E-38
Cd24a	ENSMUSG00000047139	4.3579	2.63E-25
Fst	ENSMUSG00000021765	3.8138	8.97E-44
Aldh1a2	ENSMUSG00000013584	3.5783	4.57E-09
Tgfb2	ENSMUSG00000039239	3.5448	0.00E+00
Bhlhe40	ENSMUSG00000030103	3.4108	1.52E-27
F5	ENSMUSG00000026579	3.1836	2.14E-09
Cadps	ENSMUSG00000054423	3.1473	1.14E-16
Stmn2	ENSMUSG00000027500	3.1203	5.27E-20
Prokr1	ENSMUSG00000049409	3.0486	4.90E-26
Clstn2	ENSMUSG00000032452	2.9915	5.85E-37
Odz4	ENSMUSG00000048078	2.9888	6.03E-26
H60b	ENSMUSG00000075297	2.8679	8.85E-11
Edn1	ENSMUSG00000021367	2.7636	5.30E-23
Inhbb	ENSMUSG00000037035	2.7634	4.98E-19
Pfn2	ENSMUSG00000027805	2.7620	3.84E-36
Sema3b	ENSMUSG00000057969	2.7464	0.00E+00
Slc16a2	ENSMUSG00000033965	2.7348	4.14E-24
Sp7	ENSMUSG00000060284	2.6793	7.61E-25
Padi2	ENSMUSG00000028927	2.6784	8.94E-34
Axin2	ENSMUSG00000000142	2.6729	8.47E-18
Mir206	ENSMUSG00000065559	2.6607	2.51E-35
Bmp2	ENSMUSG00000027358	2.6585	3.14E-17
Frmpd1	ENSMUSG00000035615	2.6449	0.00E+00
Col25a1	ENSMUSG00000058897	2.6381	1.59E-39
Syt13	ENSMUSG00000027220	2.6348	2.21E-34
Pla1a	ENSMUSG00000002847	2.6190	2.25E-26
Thy1	ENSMUSG00000032011	2.6052	5.27E-15
Gpm6b	ENSMUSG00000031342	2.5819	0.00E+00
Il33	ENSMUSG00000024810	2.5778	1.63E-26
Fam107a	ENSMUSG00000021750	2.5670	2.52E-26
Igfbp2	ENSMUSG00000039323	2.5480	1.70E-23
Tmem86a	ENSMUSG00000010307	2.5350	1.83E-28
Acsl3	ENSMUSG00000032883	2.5299	0.00E+00
Sema3d	ENSMUSG00000040254	2.5166	0.00E+00
Ifi205	ENSMUSG00000054203	2.4915	1.53E-14
Cyp1b1	ENSMUSG00000024087	2.4870	1.10E-13
Sema5a	ENSMUSG00000022231	2.4681	0.00E+00
Tnfrsf19	ENSMUSG00000060548	2.4339	0.00E+00
Cdc42ep2	ENSMUSG00000045664	2.4139	1.52E-30
Srgap1	ENSMUSG00000020121	2.4054	5.17E-27

Adamtsl2	ENSMUSG00000036040	2.3999	1.07E-23
Ch25h	ENSMUSG00000050370	2.3988	1.55E-16
Arl4a	ENSMUSG00000047446	2.3268	8.60E-35
Mir133b	ENSMUSG00000065480	2.3191	2.07E-36
Tmem123	ENSMUSG00000050912	2.2829	5.14E-45
Nrcam	ENSMUSG00000020598	2.2806	5.56E-32
Pcp4l1	ENSMUSG00000038370	2.2519	2.54E-11
Rasl11b	ENSMUSG00000049907	2.2502	7.85E-16
Stc2	ENSMUSG00000020303	2.2365	9.68E-26
Rxfp1	ENSMUSG00000034009	2.2284	0.00E+00
Col8a1	ENSMUSG00000068196	2.2132	2.79E-32
Fam198b	ENSMUSG00000027955	2.2092	1.08E-38
Cdk5r1	ENSMUSG00000048895	2.2071	1.99E-31
Frem2	ENSMUSG00000037016	2.2030	2.97E-38
Grik1	ENSMUSG00000022935	2.1871	4.65E-10
Baiap2l1	ENSMUSG00000038859	2.1769	0.00E+00
Tbc1d12	ENSMUSG00000048720	2.1705	4.69E-22
Gdnf	ENSMUSG00000022144	2.1651	1.30E-17
Tspan9	ENSMUSG00000030352	2.1586	2.56E-41
Gadd45b	ENSMUSG00000015312	2.1553	5.14E-30
Adamts5	ENSMUSG00000022894	2.1533	2.85E-34
Dll1	ENSMUSG00000014773	2.1360	7.68E-37
Ube2e2	ENSMUSG00000058317	2.1360	3.73E-36
2900062L11Rik	ENSMUSG00000048040	2.1217	6.25E-24
Nav3	ENSMUSG00000020181	2.1087	0.00E+00
Slc7a7	ENSMUSG00000000958	2.1046	1.11E-24
Tgm2	ENSMUSG00000037820	2.1044	2.56E-13
P4ha2	ENSMUSG00000018906	2.0864	4.44E-27
Itgb1bp3	ENSMUSG00000004939	2.0798	2.60E-32
Nkd1	ENSMUSG00000031661	2.0727	3.34E-19
Fgf9	ENSMUSG00000021974	2.0643	1.59E-33
AI464131	ENSMUSG00000046312	2.0608	3.48E-39
Scn5a	ENSMUSG00000032511	2.0582	9.14E-42
Ror1	ENSMUSG00000035305	2.0536	5.40E-16
5530400B01Rik	ENSMUSG00000043186	2.0516	8.36E-26
Gprc5c	ENSMUSG00000051043	2.0474	0.00E+00
Gadd45g	ENSMUSG00000021453	2.0467	7.13E-38
Ache	ENSMUSG00000023328	2.0366	1.07E-23
Epha3	ENSMUSG00000052504	2.0283	3.85E-17
Lmcd1	ENSMUSG00000057604	2.0279	7.66E-34
Pak3	ENSMUSG00000031284	2.0221	9.36E-38
Rab40b	ENSMUSG00000025170	2.0141	1.53E-23
Slc10a7	ENSMUSG00000031684	0.4996	3.81E-37
Gm5465	ENSMUSG00000075502	0.4994	4.84E-08
6720489N17Rik	ENSMUSG00000072066	0.4994	5.89E-09
Slc8a1	ENSMUSG00000054640	0.4962	1.01E-38
Gpr137b-ps	ENSMUSG00000075118	0.4961	9.46E-20
Snai2	ENSMUSG00000022676	0.4956	2.74E-40

Ska1	ENSMUSG00000036223	0.4956	3.73E-07
Vrk2	ENSMUSG00000064090	0.4952	5.63E-36
Bard1	ENSMUSG00000026196	0.4949	9.19E-10
Slc27a3	ENSMUSG00000027932	0.4941	2.09E-12
D330028D13Rik	ENSMUSG00000047115	0.4939	6.38E-11
Syce2	ENSMUSG00000003824	0.4935	2.75E-24
Mdc1	ENSMUSG00000061607	0.4921	3.32E-25
Avpr1a	ENSMUSG00000020123	0.4908	2.10E-14
Suv39h2	ENSMUSG00000026646	0.4893	5.28E-17
Hhip	ENSMUSG00000064325	0.4892	6.24E-14
Sh2d3c	ENSMUSG00000059013	0.4889	3.74E-15
E2f7	ENSMUSG00000020185	0.4888	2.42E-19
Zranb3	ENSMUSG00000036086	0.4876	2.00E-17
Prkcb	ENSMUSG00000052889	0.4875	1.56E-26
Pold1	ENSMUSG00000038644	0.4869	3.12E-17
Nid1	ENSMUSG00000005397	0.4861	1.48E-42
Syne2	ENSMUSG00000063450	0.4858	2.55E-11
Ndst3	ENSMUSG00000027977	0.4857	7.34E-16
Chaf1a	ENSMUSG00000002835	0.4846	1.35E-18
Efna5	ENSMUSG00000048915	0.4841	1.80E-27
Tmem48	ENSMUSG00000028614	0.4839	0.00E+00
Fads1	ENSMUSG00000010663	0.4836	4.67E-46
Cx3cl1	ENSMUSG00000031778	0.4836	8.50E-22
Apln	ENSMUSG00000037010	0.4832	5.31E-10
Topbp1	ENSMUSG00000032555	0.4829	1.68E-35
Rragb	ENSMUSG00000041658	0.4816	7.64E-13
4632419I22Rik	ENSMUSG00000085208	0.4791	1.26E-25
Rfc5	ENSMUSG00000029363	0.4751	1.70E-13
Vcam1	ENSMUSG00000027962	0.4750	0.00E+00
Msh2	ENSMUSG00000024151	0.4744	1.24E-28
Cyyr1	ENSMUSG00000041134	0.4722	8.64E-14
Prps2	ENSMUSG00000025742	0.4720	1.61E-41
Gm12181	ENSMUSG00000082250	0.4717	3.92E-18
Zfp125	ENSMUSG00000069755	0.4712	3.74E-45
Ckap2l	ENSMUSG00000048327	0.4710	8.67E-28
Dnajc9	ENSMUSG00000021811	0.4696	6.35E-27
Steap1	ENSMUSG00000015652	0.4686	5.44E-25
Fanca	ENSMUSG00000032815	0.4672	6.48E-20
Tmem171	ENSMUSG00000052485	0.4667	9.59E-13
Pla2g4a	ENSMUSG00000056220	0.4655	0.00E+00
Hn1l	ENSMUSG00000024165	0.4654	7.74E-29
BC048355	ENSMUSG00000040658	0.4650	2.72E-21
Bend6	ENSMUSG00000042182	0.4620	6.34E-31
1700007K13Rik	ENSMUSG00000026831	0.4610	7.80E-16
Dck	ENSMUSG00000029366	0.4607	1.28E-20
Myo1b	ENSMUSG00000018417	0.4606	8.41E-34
Mertk	ENSMUSG00000014361	0.4605	1.35E-21
9030425E11Rik	ENSMUSG00000032024	0.4599	2.99E-16

Cntln	ENSMUSG00000038070	0.4589	1.12E-28
Msh6	ENSMUSG00000005370	0.4569	5.17E-25
Polq	ENSMUSG00000034206	0.4561	2.42E-14
Cdc7	ENSMUSG00000029283	0.4553	2.74E-12
Usp1	ENSMUSG00000028560	0.4553	3.83E-19
Srd5a1	ENSMUSG00000021594	0.4539	4.84E-17
Gsg2	ENSMUSG00000050107	0.4536	4.24E-12
Blm	ENSMUSG00000030528	0.4528	5.95E-40
Cit	ENSMUSG00000029516	0.4525	1.26E-25
Haus6	ENSMUSG00000038047	0.4507	3.27E-38
Prrg4	ENSMUSG00000027171	0.4497	1.60E-32
Pask	ENSMUSG00000026274	0.4483	1.34E-16
Rpp25	ENSMUSG00000062309	0.4481	1.10E-05
4930534B04Rik	ENSMUSG00000061533	0.4467	3.74E-13
Ptgs1	ENSMUSG00000047250	0.4461	3.18E-32
Mme	ENSMUSG00000027820	0.4449	0.00E+00
Dnmt1	ENSMUSG00000004099	0.4435	0.00E+00
Etaa1	ENSMUSG00000016984	0.4422	2.54E-17
Sfxn1	ENSMUSG00000021474	0.4409	2.67E-34
Ccne1	ENSMUSG00000002068	0.4405	1.99E-42
Dscc1	ENSMUSG00000022422	0.4400	6.59E-07
Cenpm	ENSMUSG00000068101	0.4397	5.70E-14
Cdc45	ENSMUSG00000000028	0.4397	2.85E-27
Gfra1	ENSMUSG00000025089	0.4393	5.63E-25
Donson	ENSMUSG00000022960	0.4381	4.55E-19
Mcm2	ENSMUSG00000002870	0.4379	1.54E-36
Mcm8	ENSMUSG00000027353	0.4365	2.84E-28
Cenpp	ENSMUSG00000021391	0.4354	2.14E-15
Olfr1372-ps1	ENSMUSG00000084141	0.4351	1.92E-08
Eps8	ENSMUSG00000015766	0.4315	0.00E+00
Nmnat2	ENSMUSG00000042751	0.4311	9.30E-18
Dmp1	ENSMUSG00000029307	0.4301	6.53E-14
Fat4	ENSMUSG00000046743	0.4299	1.34E-18
Cenpl	ENSMUSG00000026708	0.4276	9.45E-16
Atad5	ENSMUSG00000017550	0.4274	7.09E-26
Ccdc34	ENSMUSG00000027160	0.4266	9.32E-26
Nsl1	ENSMUSG00000062510	0.4260	1.13E-11
Rfc4	ENSMUSG00000022881	0.4252	3.74E-44
Vav3	ENSMUSG00000033721	0.4248	2.77E-43
Gas7	ENSMUSG00000033066	0.4246	6.77E-14
Fam19a5	ENSMUSG00000054863	0.4217	1.04E-26
Smc4	ENSMUSG00000034349	0.4203	0.00E+00
Spc24	ENSMUSG00000074476	0.4184	1.54E-13
6720463M24Rik	ENSMUSG00000022070	0.4181	1.88E-21
Trim59	ENSMUSG00000034317	0.4172	1.05E-32
Cxcl13	ENSMUSG00000023078	0.4167	3.64E-12
Orc1	ENSMUSG00000028587	0.4146	4.76E-09
Fads2	ENSMUSG00000024665	0.4130	8.53E-31

4930422G04Rik	ENSMUSG00000051278	0.4129	1.79E-18
Wdr76	ENSMUSG00000027242	0.4125	3.14E-22
Lonrf3	ENSMUSG00000016239	0.4077	2.00E-10
Mcm7	ENSMUSG00000029730	0.4072	7.01E-24
Ces2g	ENSMUSG00000031877	0.4068	3.56E-09
Prim2	ENSMUSG00000026134	0.4063	1.01E-33
Mcm4	ENSMUSG00000022673	0.4063	2.02E-41
Wee1	ENSMUSG00000031016	0.4059	5.37E-34
2610021K21Rik	ENSMUSG00000021176	0.4059	7.24E-12
Sass6	ENSMUSG00000027959	0.4056	7.96E-39
Troap	ENSMUSG00000032783	0.4054	2.42E-18
Mybl2	ENSMUSG00000017861	0.4037	4.73E-10
Gtse1	ENSMUSG00000022385	0.4032	5.34E-33
Abcb1b	ENSMUSG00000028970	0.4019	3.15E-12
Tacc3	ENSMUSG00000037313	0.3993	1.27E-32
Cdc6	ENSMUSG00000017499	0.3983	6.43E-10
Ppil5	ENSMUSG00000034883	0.3978	2.70E-09
Arhgap11a	ENSMUSG00000041219	0.3933	4.53E-36
Cdk1	ENSMUSG00000019942	0.3933	6.80E-29
Tipin	ENSMUSG00000032397	0.3929	1.78E-35
Cd200	ENSMUSG00000022661	0.3914	4.76E-20
Rgs5	ENSMUSG00000026678	0.3875	4.67E-46
Dut	ENSMUSG00000027203	0.3866	5.17E-24
Iqgap3	ENSMUSG00000028068	0.3859	2.05E-17
Sema6d	ENSMUSG00000027200	0.3851	0.00E+00
Ncapd2	ENSMUSG00000038252	0.3840	6.06E-32
Lmnb1	ENSMUSG00000024590	0.3837	2.37E-26
Ccdc18	ENSMUSG00000056531	0.3836	8.69E-14
Adcyap1r1	ENSMUSG00000029778	0.3830	7.82E-30
Rbl1	ENSMUSG00000027641	0.3813	0.00E+00
Ngf	ENSMUSG00000027859	0.3806	2.31E-15
C3ar1	ENSMUSG00000040552	0.3790	6.68E-11
Pde3b	ENSMUSG00000030671	0.3790	6.74E-28
Fam54a	ENSMUSG00000019992	0.3789	1.58E-13
Hist1h1a	ENSMUSG00000049539	0.3781	4.59E-23
Dhfr	ENSMUSG00000021707	0.3776	1.35E-24
BC030867	ENSMUSG00000034773	0.3769	5.59E-12
Espl1	ENSMUSG00000058290	0.3753	3.02E-11
Eme1	ENSMUSG00000039055	0.3752	9.35E-14
Cdca3	ENSMUSG00000023505	0.3748	3.89E-19
Zdhhc2	ENSMUSG00000039470	0.3742	8.95E-20
Mns1	ENSMUSG00000032221	0.3732	4.73E-18
Hist1h4f	ENSMUSG00000069274	0.3708	3.89E-08
Fanci	ENSMUSG00000039187	0.3691	7.67E-22
Mms22l	ENSMUSG00000045751	0.3682	1.49E-31
Slc5a3	ENSMUSG00000089774	0.3681	3.75E-35
Rad51ap1	ENSMUSG00000030346	0.3667	1.89E-16
Cenpw	ENSMUSG00000075266	0.3662	1.61E-05

Gas2l3	ENSMUSG00000074802	0.3658	7.41E-35
Fbxo48	ENSMUSG00000044966	0.3655	3.74E-09
Mad2l1	ENSMUSG00000029910	0.3645	3.21E-36
Fam83d	ENSMUSG00000027654	0.3635	6.01E-30
Ercc6l	ENSMUSG00000051220	0.3619	1.99E-11
Ckap2	ENSMUSG00000037725	0.3611	8.99E-25
4632434I11Rik	ENSMUSG00000030641	0.3609	3.95E-34
Hist2h2bb	ENSMUSG00000050936	0.3591	4.44E-15
Rad54b	ENSMUSG00000078773	0.3572	2.51E-21
Cdkn3	ENSMUSG00000037628	0.3566	7.20E-16
Pcsk1	ENSMUSG00000021587	0.3561	2.55E-08
Dbf4	ENSMUSG00000002297	0.3548	1.74E-43
Clspn	ENSMUSG00000042489	0.3534	7.43E-12
Arhgap19	ENSMUSG00000025154	0.3528	8.19E-26
Mcm5	ENSMUSG00000005410	0.3525	7.31E-31
Smc2	ENSMUSG00000028312	0.3522	2.60E-29
Ccnb2	ENSMUSG00000032218	0.3521	4.29E-35
Nr4a2	ENSMUSG00000026826	0.3516	4.53E-36
Rps6ka6	ENSMUSG00000025665	0.3507	6.50E-13
Incenp	ENSMUSG00000024660	0.3505	4.63E-30
Hist1h1b	ENSMUSG00000058773	0.3504	3.76E-30
Dna2	ENSMUSG00000036875	0.3473	1.69E-20
Lig1	ENSMUSG00000056394	0.3468	4.36E-22
Mcm10	ENSMUSG00000026669	0.3462	4.39E-20
Cdca7	ENSMUSG00000055612	0.3459	4.96E-14
Fignl1	ENSMUSG00000035455	0.3447	7.77E-18
Rad54l	ENSMUSG00000028702	0.3442	7.23E-19
Cdca7l	ENSMUSG00000021175	0.3439	3.18E-19
Cldn1	ENSMUSG00000022512	0.3439	9.43E-05
Prc1	ENSMUSG00000038943	0.3420	3.76E-33
Gm8773	ENSMUSG00000073234	0.3420	7.39E-10
Ect2	ENSMUSG00000027699	0.3413	2.37E-36
Entpd1	ENSMUSG00000048120	0.3399	3.28E-07
Slc43a3	ENSMUSG00000027074	0.3372	2.19E-33
Foxm1	ENSMUSG00000001517	0.3366	7.43E-20
Cenpq	ENSMUSG00000023919	0.3346	1.01E-29
Aurka	ENSMUSG00000027496	0.3339	4.44E-31
Racgap1	ENSMUSG00000023015	0.3324	7.18E-32
Kcnh5	ENSMUSG00000034402	0.3316	3.09E-12
Gins1	ENSMUSG00000027454	0.3312	2.63E-17
Plk4	ENSMUSG00000025758	0.3307	4.73E-41
Cenpa	ENSMUSG00000029177	0.3305	2.64E-24
Pole2	ENSMUSG00000020974	0.3292	6.12E-34
Gzmd	ENSMUSG00000059256	0.3285	2.13E-18
Birc5	ENSMUSG00000017716	0.3275	1.64E-18
Mcm6	ENSMUSG00000026355	0.3262	4.36E-39
Mlf1ip	ENSMUSG00000031629	0.3240	8.55E-17
Ndc80	ENSMUSG00000024056	0.3238	5.72E-15

Diap3	ENSMUSG00000022021	0.3225	2.86E-20
Cd34	ENSMUSG00000016494	0.3206	1.57E-17
Cenpf	ENSMUSG00000026605	0.3204	4.46E-37
Fancb	ENSMUSG00000047757	0.3202	4.72E-21
Cenpn	ENSMUSG00000031756	0.3192	1.76E-33
Chaf1b	ENSMUSG00000022945	0.3170	6.82E-24
Mcm3	ENSMUSG00000041859	0.3169	1.45E-25
Prr11	ENSMUSG00000020493	0.3157	2.67E-32
Tyms	ENSMUSG00000025747	0.3155	5.74E-14
Tpx2	ENSMUSG00000027469	0.3144	2.91E-37
Epha1	ENSMUSG00000029859	0.3129	2.41E-19
Fam64a	ENSMUSG00000020808	0.3120	2.09E-14
Pde1a	ENSMUSG00000059173	0.3118	2.10E-28
Tcf19	ENSMUSG00000050410	0.3118	1.69E-16
D2Ertd750e	ENSMUSG00000027331	0.3100	1.27E-33
Cdc20	ENSMUSG00000006398	0.3092	5.71E-16
Cdca2	ENSMUSG00000048922	0.3089	2.12E-24
Brca2	ENSMUSG00000041147	0.3078	1.02E-13
Uhrf1	ENSMUSG00000001228	0.3077	3.41E-14
Sgol2	ENSMUSG00000026039	0.3077	2.65E-27
Mybl1	ENSMUSG00000025912	0.3076	1.02E-38
Tk1	ENSMUSG00000025574	0.3062	6.92E-07
Plk1	ENSMUSG00000030867	0.3059	3.64E-23
Pola1	ENSMUSG00000006678	0.3044	3.87E-30
Ccna2	ENSMUSG00000027715	0.3042	0.00E+00
Hist1h2bb	ENSMUSG00000075031	0.3025	3.58E-15
Cenpi	ENSMUSG00000031262	0.3024	3.81E-35
Gm15697	ENSMUSG00000081670	0.3021	3.63E-05
Ccne2	ENSMUSG00000028212	0.3018	9.52E-34
Rgs4	ENSMUSG00000038530	0.2989	2.42E-14
C330027C09Rik	ENSMUSG00000033031	0.2988	0.00E+00
Kif20a	ENSMUSG00000003779	0.2986	0.00E+00
Cdc25c	ENSMUSG00000044201	0.2983	4.12E-16
Kif22	ENSMUSG00000030677	0.2982	2.00E-22
Ncaph	ENSMUSG00000034906	0.2972	7.40E-40
Kif23	ENSMUSG00000032254	0.2959	0.00E+00
Slc14a1	ENSMUSG00000059336	0.2951	3.58E-13
Mki67	ENSMUSG00000031004	0.2939	5.76E-42
Aspm	ENSMUSG00000033952	0.2925	2.66E-21
Stil	ENSMUSG00000028718	0.2923	3.93E-29
Cdca5	ENSMUSG00000024791	0.2922	1.89E-11
Ccnf	ENSMUSG00000072082	0.2920	2.14E-31
Oip5	ENSMUSG00000072980	0.2904	1.23E-16
Gm12387	ENSMUSG00000084220	0.2895	1.65E-11
Dlgap5	ENSMUSG00000037544	0.2891	1.59E-31
Wdhd1	ENSMUSG00000037572	0.2883	0.00E+00
4930547N16Rik	ENSMUSG00000035365	0.2877	1.49E-24
Spag5	ENSMUSG00000002055	0.2875	5.39E-20

Atad2	ENSMUSG00000022360	0.2875	1.38E-31
Nek2	ENSMUSG00000026622	0.2871	6.63E-20
Bub1b	ENSMUSG00000040084	0.2869	0.00E+00
Brca1	ENSMUSG00000017146	0.2860	1.20E-28
Aurkb	ENSMUSG00000020897	0.2848	5.20E-27
Zwilch	ENSMUSG00000032400	0.2834	2.16E-41
Cdca8	ENSMUSG00000028873	0.2834	6.75E-21
Kntc1	ENSMUSG00000029414	0.2833	0.00E+00
Melk	ENSMUSG00000035683	0.2831	8.14E-24
Mastl	ENSMUSG00000026779	0.2822	1.01E-41
5730590G19Rik	ENSMUSG00000046591	0.2801	1.23E-35
Chek1	ENSMUSG00000032113	0.2772	1.00E-30
Nusap1	ENSMUSG00000027306	0.2755	8.76E-36
Brip1	ENSMUSG00000034329	0.2752	1.35E-22
Fancd2	ENSMUSG00000034023	0.2750	2.66E-22
Top2a	ENSMUSG00000020914	0.2745	0.00E+00
Hist1h2ab	ENSMUSG00000061615	0.2720	1.10E-10
Trip13	ENSMUSG00000021569	0.2715	3.12E-22
Kif11	ENSMUSG00000012443	0.2710	0.00E+00
Sgol1	ENSMUSG00000023940	0.2686	3.42E-30
Apcdd1	ENSMUSG00000071847	0.2684	8.32E-15
Dtl	ENSMUSG00000037474	0.2684	8.25E-33
Scn9a	ENSMUSG00000075316	0.2674	2.06E-11
Spc25	ENSMUSG00000005233	0.2672	1.10E-27
Prim1	ENSMUSG00000025395	0.2659	1.47E-24
Reln	ENSMUSG00000042453	0.2630	0.00E+00
Exo1	ENSMUSG00000039748	0.2602	5.57E-16
Kif18a	ENSMUSG00000027115	0.2592	3.53E-25
Gen1	ENSMUSG00000051235	0.2579	1.16E-29
Hmmr	ENSMUSG00000020330	0.2570	0.00E+00
Shcbp1	ENSMUSG00000022322	0.2556	3.54E-25
Hells	ENSMUSG00000025001	0.2539	7.35E-33
Kif20b	ENSMUSG00000024795	0.2534	9.25E-24
Kif18b	ENSMUSG00000051378	0.2522	3.03E-24
Fam111a	ENSMUSG00000024691	0.2521	6.19E-31
Pole	ENSMUSG00000007080	0.2506	0.00E+00
Slfn9	ENSMUSG00000069793	0.2496	1.55E-13
Kif4	ENSMUSG00000034311	0.2487	1.50E-22
F630043A04Rik	ENSMUSG00000021965	0.2474	2.15E-27
Cenph	ENSMUSG00000045273	0.2472	9.52E-17
Ttk	ENSMUSG00000038379	0.2457	1.89E-26
Cenpe	ENSMUSG00000045328	0.2456	0.00E+00
Nuf2	ENSMUSG00000026683	0.2447	2.79E-32
Ncapg2	ENSMUSG00000042029	0.2435	2.70E-29
Kif15	ENSMUSG00000036768	0.2425	2.15E-29
Casc5	ENSMUSG00000027326	0.2416	4.08E-29
Kif2c	ENSMUSG00000028678	0.2416	2.03E-32
Neil3	ENSMUSG00000039396	0.2394	4.09E-26

Cep55	ENSMUSG00000024989	0.2377	1.66E-27
AC087117.1	ENSMUSG00000091747	0.2373	9.13E-26
Rad51	ENSMUSG00000027323	0.2373	2.29E-20
Cenpk	ENSMUSG00000021714	0.2328	5.40E-25
Ncapg	ENSMUSG00000015880	0.2262	0.00E+00
Pbk	ENSMUSG00000022033	0.2259	1.06E-13
Fbxo5	ENSMUSG00000019773	0.2199	2.15E-13
Bub1	ENSMUSG00000027379	0.2199	7.56E-34
C79407	ENSMUSG00000047534	0.2127	2.93E-35
Esco2	ENSMUSG00000022034	0.1982	1.07E-27
Cxcl12	ENSMUSG00000061353	0.1947	1.30E-30
